# Efficacy of betahistine in counteracting second-generation antipsychotics-induced weight gain: A meta-analysis with trial sequential analysis

**DOI:** 10.1192/j.eurpsy.2023.685

**Published:** 2023-07-19

**Authors:** Y. Soliman, A. Azeez, W. Chibani, A. Mamdouh, B. Elawfi, A. M. Sharkawy, O. A. Abdelwahab, R. U. Awan

**Affiliations:** ^1^Faculty of Medicine, Assiut University, Assiut; ^2^Faculty of Medicine, Tanta University, Almahalla Alkubrah; ^3^Faculty of Medicine, Cairo University, Cairo; ^4^Faculty of Medicine, Misr University for Science and Technology, Giza, Egypt; ^5^Faculty of Medicine, Sana’a University, Sana’a, Yemen; ^6^Faculty of Medicine, South Valley University, Qena; ^7^Faculty of Medicine, Al-Azhar University, Cairo, Egypt; ^8^Ochsner Health System, Mississippi, United States

## Abstract

**Introduction:**

Despite being effective in schizophrenia, second-generation antipsychotics are potent histamine-H1 receptor antagonists associated with weight gain. Histaminergic agonists can potentially counteract the weight gain effects of antipsychotics. Betahistine is a centrally acting histamine-H1 agonist and, therefore, may reduce antipsychotic-induced weight gain, but it has never been examined in a meta-analysis.

**Objectives:**

This meta-analysis aims to examine the efficacy of betahistine in counteracting the weight gain effects of antipsychotics.

**Methods:**

We searched PubMed, Scopus, Web of Science, and Cochrane Controlled Register of Trials (CENTRAL) for all relevant trials. We used Hedges’ g with its confidence interval as our effect size to correct for the small sample size. The primary outcomes of this study were changes in weight and body mass index (BMI). Changes in insulin resistance and lipid parameters were secondary outcomes.

**Results:**

165 studies were included in the title/abstract screening, and 5 studies with 217 patients were finally included. Betahistine led to statistically significant changes in weight (Hedges’ g -1.13, 95% CI [-1.66, -0.60], p < 0001), BMI (Hedges’ g -1.64, 95% CI [-2.39, -0.89], p < 0.0001), and waist circumference (Hedges’ g -0.98, 95% CI [-1.47, -0.49], p < 0001). Nevertheless, betahistine did not lead to any significant changes in fasting glucose (Hedges’ g 0.02, 95% CI [-0.41, 0.44], p = 0.94) or insulin levels (Hedges’ g -0.07, 95% CI [-1.78, 1.64], p = 0.94).

**Conclusions:**

Betahistine is an effective add-on treatment for second-generation antipsychotics to counteract weight gain experienced with these medications. Further trials are recommended to examine its effect on blood lipids and side effects.

**Disclosure of Interest:**

None Declared

